# Effectiveness of straight arm traction versus operative treatment for displaced paediatric supracondylar humerus fractures: a randomised single-blind controlled, a non-inferiority trial—the STOPUS trial

**DOI:** 10.1186/s13063-025-08890-y

**Published:** 2025-06-23

**Authors:** Mengistu Gebreyohanes Mengesha, Ephrem Gebrehana Adem, Claude F. Martin, William J. Harrison

**Affiliations:** 1 Hawassa University Comprehensive Specialised Hospital, Hawassa, Ethiopia; 2 Ethiopian Society of Trauma and Orthopaedics, Addis Ababa, Ethiopia; 3 AO Alliance, Chur, Switzerland; 4 Countess of Chester NHS Foundation Trust, Chester, England

**Keywords:** Supracondylar humeral fracture, Fracture, Paediatric, Operative, Non-operative, Cost-effective, Low- and middle-income country

## Abstract

**Background:**

Supracondylar humeral fractures are common injuries in children and can be associated with high morbidity and lead to lifelong disability. The method of treatment affects the risk of complications and potentially the functional outcome. Closed reduction and percutaneous pinning CRPP has become the most widely used treatment method in high-income countries. In the current literature, both CRPP and lateral straight arm traction have been shown to give good results and reasonable levels of complications. The two methods have never been tested against each other in a randomised trial. Furthermore, these methods have not been analysed prospectively in the low and middle income context.

**Methods:**

The study will be a prospective randomised trial comparing lateral straight arm traction LSAT against CRPP. Recruitment will be at 8 large referral hospitals in Ethiopia. Based on non-inferiority power calculation, we plan to recruit 220 patients.

The principal outcome measure will be the PROMIS parent proxy upper extremity short form 8a score version 3.0 at 12 months.

The secondary outcome measures will be the Flynn’s criteria; complications; PROMIS parent proxy upper extremity short form 8a score version 3.0 at 6 months; PROMIS parent proxy global health 7 + 2 score version 3.0 administered at 6 and 12 months; and economic analysis of hospital costs for the two treatment modalities.

**Discussion:**

Supracondylar humeral fractures are common and serious injuries which occur frequently in LMICs where often half the population are under 18 years of age. Such countries have limited capacity for fracture care. Finding solutions which may avoid referral and operative intervention is paramount in developing access to timely and affordable care for all people. If LSAT is non-inferior to CRPP, then children can be treated locally in a cost-effective manner with avoidance of disability.

**Trial registration:**

ISRCTN Ref 62164933. Registered on 25 July 2024.

## Administrative information

Note: the numbers in curly brackets in this protocol refer to SPIRIT checklist item numbers. The order of the items has been modified to group similar items (see http://www.equator-network.org/reporting-guidelines/spirit-2013-statement-defining-standard-protocol-items-for-clinical-trials/).

**Table:** 

Title {1}	Effectiveness of Straight Arm Traction versus Operative Treatment for displaced paediatric supracondylar humerus fractures: a randomised single blind controlled non-inferiority trial—the STOPUS trial
Trial registration {2a and 2b}.	ISRCTN62164933 ISRCTN is approved for the WHO
Protocol version {3}	Protocol 1; 01/08/2024
Funding {4}	AO Alliance Foundation https://ao-alliance.org/
Author details {5a}	Mengistu G Mengesha, Hawassa University Comprehensive Specialised hospital Ephrem G Adem, Ethiopian Society of Trauma and Orthopaedics Claude F Martin Jr, AO Alliance William J Harrison, Countess of Chester NHS Foundation Trust
Name and contact information for the trial sponsor {5b}	Dr Claude Martin Jr, AO Alliance cmartin@ao-alliance.org
Role of sponsor {5c}	AO Alliance Foundation is funding the study. Dr Claude Martin Jr is Managing Director of the AOA. Prof William J Harrison is Africa Medical Director for the AOA. They have inputted to the study design and will support the write up. AOA is a developmental NGO with no commercial partner or interest.

## Introduction

### Background and rationale {6a}

Research question: “Research question: Is treatment of displaced paediatric supracondylar fractures using lateral straight arm traction LSAT non-inferior to operative treatment with closed reduction and percutaneous pinning CRPP in the low- and middle-income country LMIC context?

A supracondylar fracture is a break in the humerus (upper arm bone) occurring up to two inches (5 cm) above the elbow joint. The condyles of the humerus are the two rounded prominences at the end of the bone that are part of the elbow joint. Supracondylar fractures are the most common form of elbow fracture in children, with an annual incidence between 60 and 177 per 100,000 children [1, 2]; these fractures are rare in adults. Supracondylar elbow fractures can occur throughout childhood but are most common in children between 5 and 6 years of age [3]. Several epidemiological studies have reported little gender difference in the incidence of these injuries [1–3]. The typical mechanism of injury is a fall onto an outstretched hand or less commonly a direct fall onto a flexed (bent) elbow [4]. Only 1% of supracondylar fractures are open, with an associated wound providing a direct route for contamination of the bone ends; the remaining 99% are closed (with no open wound). Open fractures require urgent surgical treatment to reduce the risk of serious infection [1]. Successful treatment of a supracondylar elbow fracture in a child leads to normal function with no lasting disability. However, complications of this injury can occur because of the initial injury or because of problems during treatment. The major blood vessels and nerves in the arm can be damaged during the injury, the operation, or because of compartment syndrome. The last mentioned is a rare complication where there is excessive swelling in the arm, increasing pressure in the arm, and compressing the small blood vessels; this can cause permanent damage to the muscles and nerves if not identified and treated promptly. The other significant complication relates to the alignment of the arm. This can arise because of malunion (the bone healing up in the wrong position after injury), or because of altered growth around the elbow. Children may end up with an arm with cubitus varus (the forearm pointing towards the midline), cubitus valgus (excessive bending away from the midline), or rotational abnormalities. Cubitus varus is commonly unsightly and can be associated with reduced function of the limb. To identify and try to prevent these serious complications, an adequate history and examination to identify nerve or blood vessel compromise are essential when assessing children with this fracture. Clinical findings along with fracture pattern and the degree of displacement on x-ray can be used to predict the risk of complications. In displaced supracondylar fractures, the reported rate of vascular injury ranges from 5 to 31% and nerve injury at presentation from 5 to 15% [2–5]. Ninety-five per cent of supracondylar fractures are extension-type injuries, where the elbow has been over-extended, and the elbow moves backwards in relation to the humerus. Five per cent are flexion type injuries, where the elbow has been over-bent, and the elbow has been pushed forwards in relation to the humerus; these are typically more unstable and more challenging to treat. There are two commonly used classifications for describing the radiographic appearance of supracondylar fractures. The Gartland classification has been used extensively to describe these injuries and is useful for identification of type I (undisplaced) and type III (completely displaced) fractures [6]. Type II fractures (intact posterior cortex) may rotate or have angulation and therefore represent a heterogeneous group of fractures. The Gartland classification has been modified to further describe type II fractures based on the presence of rotation [7] and expanded to include fractures with no intact periosteum. Alternatively, the Arbeitsgemeinschaft für Osteosynthesefragengroup (German for Association for the Study of Internal Fixation) or AO classification offers improved clarity in describing the expected stability of these fractures [8].

#### Statement of the problem

There are several treatments available for supracondylar fractures, the choice of which will depend on factors including the severity of the injury, nerve and vascular status of the limb, and surgeon’s preference. Recent studies have demonstrated divergence of surgeon preference practice from national guidelines, with unclear consequences for long-term recovery [9]. Non-surgical treatments of undisplaced fractures involve immobilisation of the arm in a sling, splint, or cast for 3 to 5 weeks to keep the elbow in the correct position. A cast is typically constructed of plaster of Paris or a synthetic resin and extends from above the elbow to the forearm or the hand. Minimally displaced fractures can be treated in several ways. Some displacement can be accepted, relying on the child’s ability to remodel (grow the bone out straight), or it can be reduced. Reduction (repositioning the broken bone back to the normal alignment) commonly involves bending the elbow up to bring the bone into the normal position and then treating it in a sling, cast, or splint as above. Some displaced fractures can be treated with traction. Traction can be applied as skin traction (where the traction is strapped to the child’s arm) or skeletal traction (where the traction is applied directly to the bone using a screw, pin, or wire). The non-surgical method of skin traction is more common than skeletal traction, which requires an operation to insert the screw, pin or wire. In both methods, the limb is kept straight whilst it heals using weights attached through pulleys or overhead traction. The arm can be positioned to the side of the child (Dunlop traction) or above the child (overhead traction). Treatment with traction requires a prolonged hospital stay. Surgical management of supracondylar fractures in children is achieved following general anaesthesia, using a closed or open technique to reduce (realign) the fractured bone. Stabilisation of the fracture is most often performed using percutaneous wires: smooth metal Kirschner wires (K-wires) are placed through the skin. Two or more wires are inserted, depending on the stability of the break. A crossed K-wire technique will have an entry point on either side of the elbow forming an ‘X’ on x-ray, whereas a lateral wire divergent technique has the wires inserted only from the outside part of the elbow. Children treated in this manner are commonly treated in cast to protect the wires until wire removal 3 to 4 weeks after the fixation. Alternative but rarer stabilisation techniques include the use of a plate and screws, or an external fixator, where a frame is seen on the outside of the limb, which holds the bones together whilst they heal. Minimally displaced fractures are routinely reduced in the hospital emergency department and immobilised. Very displaced fractures require a more formal reduction with significant sedation or a general anaesthetic. Most centres will perform this in the operating theatre, as fixation of the fracture can be performed at the same time. Some centres will attempt reduction with sedation in the hospital emergency department to reduce the delay to reduction. Most severely displaced fractures are unstable and so require fixation in theatre even after an initial reduction. This leads to two procedures instead of one. Waiting for theatre availability often adds a significant delay that can cause an increase in nerve or vascular damage.

#### Significance of the study

A Cochrane Library review in 2022 [9] looked at the evidence for the various interventions for treating supracondylar elbow fractures in children. There are several key directions for future research uncovered by this review. There was considerable heterogeneity of outcomes presented by different trials, which has limited our ability to combine all identified trials into the meta‐analyses. There is an urgent need to agree and harmonise outcomes, and the tools to measure them, to ensure all trials measure and report key results that can then be pooled. This can be achieved with the adoption of a core outcome set to encourage harmonisation of outcome reporting [10]. Additional preparatory work in the anticipation of future trials would be to undertake validation of functional, emotional, and quality of life scores for children with supracondylar fractures to ensure validity, reliability, and interpretability of patient‐reported scores.

A comparison of traction and surgical fixation would be of greater benefit, particularly for application in resource‐poor environments around the world or for settings where access to safe anaesthesia is limited. Any further comparison of retrograde crossed and retrograde lateral wires should be high quality and capture outcomes using validated patient‐reported scores with adequate follow‐up. As the key uncertainty is if the increased loss of reduction has any clinical significance, such a trial would need to be large in scale.

#### Literature review

Supracondylar fractures of the distal humerus are the most common injury requiring intervention in children aged < 7 years and constitute 18% of the fractures sustained by those aged < 16 years [11]. Classification of such injuries is based on a system initially described by Gartland [6]. Undisplaced Gartland type I fractures are typically managed with cast immobilisation, resulting in good functional outcomes and are not the focus of this study [12, 13] Historically, closed reduction and casting provided the mainstay of treatment; however, rates of Volkmann’s ischaemic contracture were high [14]. In the 1920 s, Dunlop began treating displaced supracondylar fractures with traction [15], and by doing so, successfully reduced the frequency of serious complications [15]. However, long hospital stays, and the inherent associated costs led to a shift in favour of operative management [16]. In many LMICs, surgeons often lack access to resources or expertise for operative management [17]. Consequently, conservative management remains the norm [18]. In high-income countries, where surgical treatment is routine, options include open reduction and internal fixation (ORIF) or open/closed reduction with fixation by percutaneous Kirschner wires. The American Association of Orthopaedic Surgeons (AAOS) recommends closed reduction with pin fixation for all patients with displaced injuries [12]. The British Orthopaedic Association Standards for Trauma (BOAST 11) recommends early surgical treatment for these injuries [19]. There is no Level-1 evidence available comparing the outcomes of operative versus non-operative management of these fractures.

A recent review of the published evidence regarding the outcome of conservatively managed displaced supracondylar fractures in children has shown that current evidence for the management of displaced supracondylar fractures is inconclusive. It appears that closed reduction and casting may be utilised in the first instance with positive results, with the option of percutaneous pinning in the event of failed reduction. Whereas outcomes following traction appear to be equivalent to that of percutaneous pinning, this conclusion is drawn from a limited number of studies. Despite this, the trend of managing all displaced injuries operatively within high-income settings remains unchallenged. Where resources allow, operative intervention is now regarded as the gold standard management for Gartland II and III injuries [12]. The British Orthopaedic Association Standards for Trauma state that displaced supracondylar fractures ‘…require early surgical treatment; ideally on the day of admission... surgical stabilisation should be with bicortical wire fixation [19].’ However, these are guidelines for resource-rich environments and cannot be translated to healthcare provision in resource-limited settings. The results of the literature review suggest that where surgical intervention is unavailable, traction remains the preferred management.

Anatomical reduction is required for percutaneous pinning to succeed. Hence, attempting the procedure without the aid of intraoperative fluoroscopy is hazardous, limiting its use to environments where such resources are available. Complications such as ulnar nerve injury, pin migration, and pin track infection are reported in the literature with rates in the range of 1.8–4.7% [20–22]. O’Hara et al. report rates of cubitus varus deformity of up to 32% when protocol and x-ray are not strictly followed with K-wire insertion [23] There is therefore a trade-off between conservative management, where possible mild malunion would result in normal function but a potential cosmetic problem, and operative intervention where the intra- and post-operative complications can be significantly worse.

It is not only the access to surgical skills and equipment that limits the use of operative intervention in LMICs. Access to anaesthesia is a problem throughout sub-Saharan Africa, where facilities to deliver safe anaesthesia to children have been reported to be as low as 13% [21]. This additional risk of operative intervention provides further insight as to why traction remains the preferred method of treatment in many countries. Loss of reduction was the only indication reported in the four studies recommending initial closed reduction with subsequent operative intervention. The disparity between outcome measures used gives the data limited transferability. Although Flynn’s criteria were used the most frequently, outcome measures in the literature are wide-ranging [22]. Flynn provides a method of analysis whereby results can be easily compared using change in carrying angle and range of motion. However, this clinical outcome is not patient-reported and can be prone to measurement bias. Changes in carrying angle, associated with a poorer score of Flynn’s criteria, may not always equate with a worse functional outcome. There is a need for a validated functional outcome measure in children, encompassing patient-reported outcome measures. One limitation of many articles in the review is the lack of transparency when allocating patients to treatment groups. It was frequently unclear how patients had been selected to be managed conservatively or operatively. Indeed, in retrospective case series, this cannot accurately be measured. Recruitment bias may well therefore have confounded several authors’ conclusions.

Cubitus varus is widely considered a cosmetic problem, usually only evident when standing in the anatomical position [24]. Review of patients with residual cubitus varus following supracondylar fracture found no functional deficit, and deformity can be corrected via planned geometric osteotomy later, if required (70). The incidence and long-term consequences of cubitus varus deformity in LMICs have not been investigated in the current literature.

Traction is well documented to result in a longer hospital stay than operative intervention. Duration of inpatient stay was in the range of 11–22 days with a median of 19 days. Two papers used length of stay as contributing factors to their cost analysis, both concluding traction was considerably more expensive than pinning [17, 25]. When considering duration of stay, theatre fees, anaesthetic fees, recovery room fees, and radiography fees, Sutton et al. and Piretto et al. calculated traction was more expensive by 142% and 179%, respectively. However, both papers were based in high-income countries, where costs of both equipment and service provision make calculations non-transferable to less economically developed nations. The use of traction for Gartland types II and III supracondylar fractures provides a safe and effective alternative to percutaneous wire fixation in the resource-poor setting. In countries where few specialist centres are managing increasingly high volumes of trauma, the benefit of such surgical intervention remains to be proven. With the correct expertise, traction can be safely applied in a local setting, avoiding the need for long-distance transfer and associated financial cost.

Currently there is no Level-1 evidence comparing percutaneous pin fixation with traction for displaced supracondylar fractures of the distal humerus in children. Drawing on conclusions from the studies reviewed, there is a suggestion that these two management options remain part of the solution with no clear recommendation.

While published studies have been unable to answer if displaced fractures should be treated with reduction and casting or by other techniques (e.g. traction or surgical fixation), we would suggest that this is not a key research priority, given the American Academy of Orthopaedic Surgeons (AAOS) and British Orthopaedic Association/British Society for Children’s Orthopaedic Surgery (BOA/BSCOS) guideline and the high treatment failure rates when casting has been used. A comparison of traction and surgical fixation is of greater benefit, particularly for application in resource‐poor environments around the world or for settings where access to anaesthesia is limited. Any further comparison of retrograde crossed and retrograde lateral wires should be high quality and capture outcomes using validated patient‐reported scores with adequate follow‐up. As the key uncertainty is if the increased loss of reduction has any clinical significance, such a trial would need to be large in scale.

Blinding participants and outcome assessors is challenging, particularly as treatments can be viewed as they are applied or removed. Children and their parents are in an ideal position to provide insight into their experience and function following an intervention, and this is unlikely to be clouded by preconceived ideas of superiority of one intervention over another. There is scope for improvements in study reporting, with prospective trial registration and publication of trial protocols to improve study transparency and confidence surrounding the quality of evidence.

### Objectives {7}

1. To test if LSAT is non inferior to CRPP as measured by the PROM
  PROMIS Parent Proxy Upper Extremity – Short Form 8av3.0 at 12 months after injury, and in the LMIC context.
2. To assess Global Health at 6 and 12 months using PROMIS
  Parent Proxy Global Health 7+2 v3.0
3. To assess complications of both LSAT and CRPP
4. Economic evaluation to compare hospital-based costs for LSAT and CRPP
5. To compare elbow function by Flynn’s criteria 3 months after LSAT/CRPP
6. To compare guardian satisfaction with treatment by LSAT/CRPP

### Trial design {8}

Prospective randomised non-inferiority trial between two established treatments. It is single-blind for the reviewers who will score the principal outcome measure.

## Methods: participants, interventions and outcomes

### Study setting {9}

The study is set in 8 referral hospitals in Ethiopia. The study sites are follows: Hawassa University Comprehensive Specialised Referral Hospital; Gondar University Hospital; Bahir Dar University Hospital; Mekele University Hospital; Black Lion Teaching Hospital, Addis Ababa; St Paul’s teaching Hospital, Addis Ababa; Alert teaching hospital, Addis Ababa; Jijiga University Hospital.

### Eligibility criteria {10}

Inclusion criteria:

- Paediatric patients (age range: 3–10 years)
- Displaced closed supracondylar humerus fractures (Gartland type IIb and III) presenting to the treating institution within 72 h of the injury
- Informed consent obtained from parents or legal guardians

Exclusion criteria:

- Associated vascular injuries (nerve injuries to be documented and will be included in the study)
- Open fractures
- Gartland I and IIa (extension without rotation) injuries
- Associated neuromuscular disorders
- Associated metabolic bone disorders
- Presence of polytrauma, ipsilateral humerus fracture, and distal radial fracture
- Flexion type of supracondylar fractures
- Refracture at the same side and site
- Limb deformity or malfunction prior to injury
- Patients unable or unwilling to attend the follow up

Study centre criteria:

- Resident-trained Trauma and Orthopaedic surgeons able to provide care.
- Working C arm image intensifier at outset of study to enable CRPP technique
- Local permission granted to conduct study

### Who will take informed consent? {26a}

The Trauma and Orthopaedic surgeons undertaking care of the patients will explain the study, provide the patient information sheet in the local language, and take informed consent using the local language understood by the guardian of the child. Parents and guardians declining to consent to the study will not be included but will still be treated in the normal way.

### Additional consent provisions for collection and use of participant data and biological specimens {26b}

Patients will consent to their anonymised data being stored electronically with password protection.

No specimens will be taken.

## Interventions

### Explanation for the choice of comparators {6b}

The two treatment modalities chosen are accepted in the literature to give excellent results, and if delivered correctly, can give a low rate of complications.

Lateral straight arm traction LSAT is a low-cost intervention which can be administered by trained orthopaedic technicians. It avoids surgery or anaesthesia but has a longer length of hospital stay.

Closed reduction and percutaneous pinning CRPP is the most popular treatment method in High Income Countries HICs. It requires an operation under general anaesthetic, with a trained T&O surgeon, and availability of a C-arm for radiographic imaging during the operation. It allows a shorter average hospital stay, with the child normally discharged from hospital within 48 h of surgery (Table 1).

**Table 1:** Schedule of enrolment interventions and assessment

	Study period						
	Enrolment	Allocation	Post allocation				Close-out
Timepoint			Within 48 h	4 weeks	3 months	6 months	12 months
Enrolment	X						
Eligibility screen	X						
Informed consent	X						
Allocation		X					
Interventions							
Closed reduction and perc pinning			X				
Lateral straight arm traction			X				
Assessments							
Flynn’s criteria				X	X		
Complications				X	X		
PROMIS scores						X	X

### Intervention description {11a}

- Percutaneous pinning CRPP:
  - Preparation: Once the fracture is reduced, percutaneous pinning involves the insertion of two metal pins (also known as Kirschner wires or K-wires) into the bone to maintain alignment and stability during the healing process.
  - Selection of pins: The surgeon selects appropriate-sized K-wires based on the patient’s age, bone size, and fracture characteristics. These pins are typically made of stainless steel or titanium and come in various diameters and lengths.
  - Insertion: Using a percutaneous approach (through the skin), the surgeon carefully inserts the K-wires across the fracture site and into the bone under fluoroscopic guidance to ensure accurate placement. The pins are driven through the skin and soft tissues and into the bone fragments.
  - Configuration: Depending on the fracture’s complexity and stability, the pins may be placed in different configurations (such as crossed from both sides or parallel from the radial side) to provide optimal stability and alignment.
  - Safe pin insertion: a pin introduced from the medial side passes close to the ulnar nerve and a small open wound is required to ensure that the nerve is not injured in this process.
  - Confirmation: Once the pins are in place, the surgeon verifies their position and alignment using imaging techniques, such as X-rays, to ensure proper fixation of the fracture fragments.
  After completing the closed reduction and percutaneous pinning, the surgical site is typically dressed, and the patient is monitored in the recovery room before being transferred to a hospital ward for further observation. Postoperative care may involve immobilisation of the elbow using a splint or cast, pain management, physical therapy, and follow-up appointments to monitor healing progress and remove the pins at 3 to 4 weeks. The goal of CRPP is to restore normal alignment and function of the elbow joint while promoting optimal healing and minimising the risk of complications.

- Lateral straight arm traction LSAT
  - The child should be placed in straight arm traction as soon as the diagnosis and treatment plan have been made.
  - Consent may be taken verbally from a parent or guardian.
  - The child is given a strong but appropriate dose of analgesia or sedation from a trained practitioner.
  - Skin traction is applied to the forearm of the injured arm from the level of the elbow crease to the wrist crease. If necessary, a crepe bandage may be applied around the forearm from distal to the elbow crease to help the skin traction to stick. However, any such bandage must NOT be bound in any tension and must be removed if the child complains of pain or that it is tight or if there are concerns about the circulation or compartments.
  - The traction cord extending from the skin traction beyond the hand is tied to a drip stand.
  - The drip stand is placed beside the bed so that the angle of the axilla is 90°.
  - The elevation of the drip stand is adjusted so that the child’s arm is elevated off the bed with the elbow almost straight. An angle of 30 to 60° should be present between the mattress and the elevated arm.
  - The elbow should not be flexed more than 30°.
  - The child is given regular oral analgesia (preferably paracetamol and ibuprofen at appropriate doses) for the first 5 days or until comfortable.
  - The traction is checked daily for circulation and movement (rock/paper/scissor/ok); and for slippage or blistering on the skin.
- Traction may be applied by any appropriate member of the T&O team. However, the traction set up must be checked by a consultant T&O surgeon within 24 h of application, or sooner if there are any problems.
- If there are any concerns regarding compartment syndrome or impaired circulation to the hand, then all circumferential bandages or dressings must be removed immediately, and the surgeon informed immediately.
- The traction should be kept there for at least 5 days, and the child should be assessed for comfort. If the swelling has all receded and the child is comfortable to flex the elbow to at least 90°, then a posterior slab should be applied with the forearm in mid prone and the elbow flexed at least 90°. Radiographs of the affected elbow AP and lateral will be taken in the cast, and if satisfactory, the child will be allowed home to attend for cast removal at 4 weeks from injury.
- If at least 5 days of the traction, if there is still swelling or the child has too much pain to flex the elbow to 90°, then straight arm traction should be continued until such a time as this becomes possible.
- If a child reaches 4 weeks in traction without qualifying for conversion to a backslap, then a fresh radiograph should be taken. If the problem is stiffness but pain is controlled and callous is present, the child can be released from traction and taught to bend the elbow, and allowed home with no backslap. No traction or back slab should be retained for more than 4 weeks since the injury.
- If at 4 weeks, there is still pain and swelling and little callous, then the surgeon must assess the situation to decide on whether to prolong traction or switch to surgical management.

### Criteria for discontinuing or modifying allocated interventions {11b}

The study design is pragmatic, and treating surgeons are permitted to intervene according to their clinical expertise and judgement. Cross-over is much more likely to occur from the non-operative LSAT to the operative CRPP, although in theory, a CRPP could be put in traction after surgery.

All cross over patients must be recorded as such, and the reasons for cross over recorded.

Likely reasons for crossover from LSAT to CRPP would be patient or guardian intolerance; skin reaction to traction material; failure to control pain; arterial or nerve compromise deemed to require surgical intervention.

### Strategies to improve adherence to interventions {11c}

Adherence will be more of an issue with the LSAT arm of the study, which has the potential to cross over.

The surgeons have been educated in application and expectations for this technique. The site PIs are also educating their colleagues and the nurses in their institutions regarding LSAT.

The patient information, counselling and consent is key to helping the children and their guardians understand what is being offered and undertaken on their behalf.

### Relevant concomitant care permitted or prohibited during the trial {11 d}

The trial is pragmatic and surgeons are able to use their clinical judgement to deliver the best possible care in the interests of the children.

One anticipated non-adherence is that in the operative CRPP group, some surgeries will end up being performed using open reduction. This may happen because closed reduction cannot be achieved, or because the C arm required for assessment of closed reduction is not available or not working. Such cases will remain in the study under the CRPP arm as randomised.

### Provisions for post-trial care {30}

The care of all patients is within the public health system of Ethiopia. Any ongoing care needs will continue to be incorporated into that health system.

### Outcomes {12}

Primary outcome: PROMIS Parent Proxy Upper Extremity – short Form 8a version 3.0, administered to patient guardian at 12 months after injury by an independent technician using Amharic or appropriate language explanation over telephone. The technician scorer will be blinded to the type of treatment received by the child and will not be permitted to ask which treatment was received.

Secondary outcomes:

1. PROMIS Parent proxy Upper Extremity – short form 8a v3.0 at 6 months
2. PROMIS Parent Proxy Global Health 7 + 2 v3.0 score at each of 6 and 12 months
3. Complications, notably nerve injury, arterial compromise, Volkmann contracture, infection, secondary intervention. Scored by surgeon assessors at 3 months post injury by face-to-face clinical review.
4. Flynn’s criteria for elbow range of movement and carrying angle assessed clinically in person by treating surgeons at 3 months after injury.
5. Patient satisfaction with treatment using 10-point visual analogue scale. Scored in person by patient guardian, administered by treating surgeon at 3 months after injury.
6. Economic analysis of hospital costs. Time and motion study at two representative hospital sites assessing both LSAT and CRPP using median hospital stays.

### Participant timeline {13}

Arrive at hospital within 72 h of injury.

Invitation to join study and informed consent process.

Treatment administered within 48 h of consent.

Discharge in plaster backslab when clinically safe as judged by treating surgeons. Minimum time for LSAT is 5 days.

Clinical outpatient review 4 weeks after injury to remove plaster and start self-mobilisation.

Clinical review 3 months after injury and discharge from clinical reviews if outcome judged satisfactory by treating surgeons.

Telephone review 6 and 12 months after injury.

### Sample size {14}

Power analysis has been performed to determine the sample size required for a non-inferiority study based on the PROMIS Upper extremity parent proxy short form 8a version 3.0 score primary outcome, with the Mean Clinically Important Difference MCID set at 3.5 to detect clinically significant differences between the two treatment groups. Initial calculations suggest 202 patients would be required (101 per group). We will aim to recruit 222 patients to the study, leaving a 10% margin of error to account for loss to follow-up. Patients will be randomly assigned to either the straight arm traction group (Group 1) or the operative treatment group (Group 2) using a computer-generated randomisation sequence. Allocation concealment will be maintained to ensure blinding.

### Recruitment {15}

We have done an analysis of case numbers presenting at the 8 included hospital sites and believe that the numbers available for recruitment exceed required numbers by 50%.

If recruitment is too slow to enable recruitment of the whole sample over 12 months, then we plan to add further centres in Ethiopia.

Our patient information leaflet has been translated into all relevant local languages. The site PIs have been educated regarding the rationale for the study and the informed consent process. They have cascaded education to their departments.

Consenting patients will be offered a small stipend to support attendance at follow-up visits.

## Assignment of interventions: allocation

### Sequence generation {16a}

Randomisation will be computer generated block randomisation to ensure equal numbers over the intended sample size of 220 patients. Randomisation will be stratified according to the Gartland 2b (rotational displacement only) and Gartland 3 (total off-end displacement) fractures as these are seen to represent separate patient sub-groups.

Ensuring equal numbers of each of these two sub-groups in each treatment arm will eliminate bias based on injury severity characteristics.

### Concealment mechanism {16b}

Following consent, the PIs will access the randomisation online, giving complete concealment.

### Implementation {16c}

The computer generation of the block randomisation will be controlled by the study overseer Dr. Mengistu Gebreyohanes Mengesha. Patients will be enrolled by the site PIs and their surgeon teams; application of treatment modality following randomisation will be by the local treating surgeon teams.

## Assignment of interventions: blinding

### Who will be blinded {17a}

The participants and care providers will not and cannot be blinded as there is an obvious visible difference between the non-operative LSAT and operative CRPP treatments.

The scoring technicians who administer the primary outcome PROMIS UE questionnaire and the secondary outcome PROMIS 7 + 2 will be blinded to which treatment the patient received. This scoring will be done by telephone, and the technicians are not part of the treating team, so at no point see the patient. They will be required not to ask which treatment was received, and to request the guardian not to volunteer which treatment had been received.

The study data analyst will also be blinded as to which treatment arm is which when analysing the results.

### Procedure for unblinding if needed {17b}

There is no instance where the scoring technicians may be required to unblind.

The treating teams are not blinded at any point and can therefore intervene for any complications or issues based on normal clinical judgement.

## Data collection and management

### Plans for assessment and collection of outcomes {18a}

The primary outcome measure is the PROMIS Pediatric Parent Proxy Short Form Upper Extremity version 3.0. At analysis, this score will be analysed as a mean for each group, and the Minimum Clinically Important Difference of 3.5 points will be applied to assess for inferiority of one or the other treatment modality.

Furthermore, we will analyse the range of scores to understand the distribution of negative outliers in each group who may have had a particularly poor outcome. This range will also be presented in the results.

The secondary outcome includes the PROMIS Parent proxy Global Health 7 + 2 PROM.

These two PROMIS will be administered by telephone to parents of the children, and data filled contemporaneously with the answers will then be entered into the STOPUS web database. The total scores will be converted into T scores according to the specific PROMIS converter tables validated for each score. The telephone scoring technicians will be specifically trained to do the PROMIS scores by telephone.

Complication data will be measured against a checklist by treating surgeons trained to assess complications. Findings will be entered by surgeons to the STOPUS database.

Parent satisfaction visual analogue scale will be scored by pointing to a laminated sheet and the score entered by the surgeon to the electronic STOPUS database.

The Flynn’s criteria will be measured using a goniometer by surgeons trained for this, and the findings will be entered to the STOPUS electronic database.

### Plans to promote participant retention and complete follow-up {18b}

Patents will be offered a small travel allowance to attend the 3-month follow-up outpatient visits.

By doing the 6-month and 12-month follow-up scores over the telephone, no face-to-face attendances will be required beyond 3 months. This will make it more affordable to families, and the risk of patient loss to follow-up is reduced.

More than one telephone contact number will be taken so as to assist in tracing patients if parents fail to answer their telephone or the number becomes unavailable.

### Data management {19}

Data will be electronically stored in a secure password protected database. Each PI will have their own personal login and password. PIs will be able to access, but not alter, their own data.

Only the study national lead will be able to access all the data.

After 3 months of recruitment, there will be a training event looking at early data and checking accuracy.

### Confidentiality {27}

Patients will be given a study number and all data will be held in a password protected database using their study number as their unique identifier.

### Plans for collection, laboratory evaluation and storage of biological specimens for genetic or molecular analysis in this trial/future use {33}

There will be no biological specimen taken or stored in this study. There will be no blood tests collected as part of the study.

## Statistical methods

### Statistical methods for primary and secondary outcomes {20a}

The data analysis will be conducted by a statistician who is blind to the treatment groups and has not been part of the clinical study.

1. Analyse the primary outcome using appropriate statistical methods, such as linear mixed-effects models or generalised estimating equations, adjusting for baseline functional status and potential confounders. The means of the 12-month PROMIS UE Proxy score will be used for assessing a difference above the MCID. Range analysis, including negative outliers, will also be assessed and presented in the results.
2. Report estimated treatment effects.
3. Compare the incidence of complications between treatment arms using appropriate statistical tests (e.g. chi-square test, Fisher’s exact test).
4. Calculate relative risks or odds ratios for complications between treatment arms with corresponding confidence intervals.
5. Explore potential treatment effect modification by conducting subgroup analyses based on relevant patient characteristics (e.g. age, fracture severity).
6. Evaluate for interaction effects between treatment assignment and subgroup variables.

Missing data handling:

1. Assess the extent of missing data and explore reasons for missingness, particularly for outcome variables.
2. Utilise appropriate methods for handling missing data, such as multiple imputation or sensitivity analyses, to assess the robustness of results.
3. Over-recruit by 10% case numbers above the power calculation to mitigate for missing data.

Sensitivity analyses:

1. Perform sensitivity analyses to examine the impact of different assumptions or analytical approaches on study results (e.g. excluding patients with incomplete follow-up, adjusting for potential confounders).
2. We intend to analyse and present results according to ‘intention to treat’ but will also undertake and present an analysis of ‘actual treatment’.

### Interim analyses {21b}

Since both treatment options are established and accepted options, and the LSAT method may be slower to achieve full recovery of function and therefore PROM, the study will be allowed to recruit the expected 220 patients unless a safety concern is raised by the PIs or the complication findings. Complication findings will be assessed at 3-month intervals or additionally if a concern is raised.

### Methods for additional analyses (e.g. subgroup analyses) {20b}

We will undertake a subgroup analysis for the two fracture patterns (Gartland 2b and Gartland 3). The randomisation is already stratified for these two slightly separate diagnoses.

The subgroup analysis will include primary and secondary outcomes using the statistical techniques described above.

### Methods in analysis to handle protocol non-adherence and any statistical methods to handle missing data {20c}

Investigate and report any protocol deviations or violations and assess their potential impact on study outcomes.

See also the section above relating to missing data.

Some protocol deviations are expected regarding cross over from non-operative LSAT to operative CRPP. This will be considered using the intention to treat and actual treatment analyses, and both will be presented.

Minor variations within the protocol arms will be noted and ignored as the aim is to be pragmatic and reflect normal variations between treating surgeons when using one or other of these two methods. Thus, all such patients will be included in the analysis.

### Plans to give access to the full protocol, participant level data and statistical code {31c}

Our full data would be available on request to relevant parties.

## Oversight and monitoring

### Composition of the coordinating centre and trial steering committee {5 d}

Our oversight team includes a Project Manager, Financial Manager, International Medical Director, National Society President, National Surgeon Manager, and the PIs.

Progress meetings, problem solving, and interim reporting and data checks will be undertaken every 3 months either in person or online.

### Composition of the data monitoring committee, its role and reporting structure {21a}

Data monitoring will be undertaken by the national Surgeon Director with reference to the International Medical Director.

Whilst the International Medical Director is a consultant for the funding body, AO Alliance Foundation, this organisation is a developmental NGO without commercial bonding and is therefore not considered to be a conflict of interest.

### Adverse event reporting and harms {22}

Assessment of complications—iatrogenic and other—forms one of the secondary outcomes of the study and will be related at 4 weeks, and 3 months. Since both treatment methods are recognised as standards of care and are in current use in Ethiopia, we consider that this is a low-risk study for adverse events. The national surgeon lead and the international Medical Director will be responsible for assessing adverse events and suspending or stopping the trial if concerns arise.

Interventions made by the oversight team will be reported in all publications.

### Frequency and plans for auditing trial conduct {23}

No independent audit is planned.

### Plans for communicating important protocol amendments to relevant parties (e.g. trial participants, ethical committees) {25}

The ethical committee and the trial registration body will be informed of any protocol amendments. The PIs will be informed and will cascade to their teams.

### Dissemination plans {31a}

We plan to disseminate results by local, national and international presentations and one or more publications in a relevant international peer-reviewed journal.

## Discussion

The study is set up with 8 participating sites, but the Trial management team will invite further sites to participate if recruitment is slow, such that the anticipated recruitment period will exceed 12 months.

### Trial status

This is protocol v1.

Recruitment started 01/08/2024.

Anticipated recruitment is 12 months ending 31/07/2025.

## Data Availability

The complete dataset will be handled by the National lead (MGM) and the International Medical Director (WJH). Hospital PIs will have access to and rights over their own hospital data.

## Abbreviations

LSAT: Lateral straight arm traction
CRPP: Closed reduction and percutaneous pinning
NGO: Non-Governmental Organisation
LMIC: Low- and middle-income country
HIC: High-income country
AO Alliance: Arbeitsgermeinshaft fur Osteosynthesefragen Alliance
T&O: Trauma and Orthopaedics
IRB: Institutional Review Board
